# Integrating genomic profiling to clinical data: assessing the impact of CD147 expression on plaque stability

**DOI:** 10.3389/fcvm.2024.1425817

**Published:** 2024-09-13

**Authors:** Yu Chen, Si Lu, Yong Ren, Jun Fan, Chun-Ping Bao, Xin Zhang, Yan-Kun Shi, Yan Wang, Li-Xia Yang

**Affiliations:** ^1^ Department of Cardiology, 920th Hospital of Joint Logistics Support Force, PLA, Kunming, China; ^2^ Department of Clinical Medical College, Dali University, Dali, China; ^3^ Department of Pulmonary and Critical Care Medicine, 920th Hospital of Joint Logistics Support Force, PLA, Kunming, China; ^4^ Department of Cardiology, First Affiliated Hospital of Kunming Medical University, Kunming, China

**Keywords:** CD147, BSG, acute coronary syndrome, unstable angina, stable angina, bioinformatics, platelets, cross-sectional study

## Abstract

**Background:**

Acute Coronary Syndrome (ACS) continues to be a leading cause of death and illness worldwide. Differentiating stable from unstable coronary plaques is essential for enhancing patient outcomes. This research investigates the role of CD147 as a biomarker for plaque stability among coronary artery disease patients.

**Methods:**

The study began with high-throughput sequencing of blood samples from six patients, divided equally between those with Stable Angina (SA) and Unstable Angina (UA), followed by bioinformatics analysis. Expanding upon these findings, the study included 31 SA patients and 30 patients with ACS, using flow cytometry to examine CD147 expression on platelets and monocytes. Additionally, logistic regression was utilized to integrate traditional risk factors and evaluate the predictive value of CD147 expression for plaque stability.

**Results:**

Initial sequencing displayed a notable difference in CD147 expression between SA and UA groups, with a significant increase in UA patients. Further analysis confirmed that elevated platelet CD147 expression was strongly associated with unstable plaques (OR = 277.81, *P* < .001), after adjusting for conventional risk factors, whereas monocyte CD147 levels did not show a significant difference.

**Conclusion:**

Elevated CD147 expression on platelets is a crucial biomarker for identifying unstable coronary artery plaques, offering insights into patient risk stratification and the development of targeted treatment strategies. This underscores the pivotal role of molecular research in understanding and managing coronary artery disease, paving the way for improved clinical outcomes.

## Introduction

Acute Coronary Syndrome (ACS) is one of the leading causes of death worldwide (1). The onset of ACS is closely associated with the rupture of unstable plaques within the coronary arteries, followed by subsequent thrombus formation (2). These unstable plaques, compared to their more structurally robust and rupture-resistant stable counterparts, are more prone to trigger acute cardiovascular events due to their thin fibrous caps and rich lipid content (3). Therefore, a deeper understanding of the characteristics of these two types of plaques is essential for the prevention of ACS.

Platelets, as a crucial component of the circulatory system, not only play a key role in maintaining internal equilibrium and regulating inflammatory responses but also promote the development of arteriosclerosis (4). While platelets have traditionally been understood to lack genomic DNA, recent findings suggest the presence of genomic DNA fragments derived from megakaryocytes in platelets (5). Despite this, platelets remain primarily reliant on stored RNA and translational mechanisms for protein synthesis, allowing them to rapidly respond to environmental stress (6).

Monocytes play a pivotal role in the formation of unstable atherosclerotic plaques. Recruited to the plaque, they differentiate into macrophages, participate in foam cell formation, produce reactive oxygen species, and contribute to proteolysis (7). These processes are critically important to plaque formation, rupture, and the pathophysiology of arteriosclerosis (8). The activation of monocytes is closely associated with the development of a prone-to-rupture plaque phenotype, leading to the progression of vulnerable and unstable plaques (9).

The Extracellular Matrix Metalloproteinase Inducer (CD147), also known as Basigin (BSG), is a transmembrane protein belonging to the immunoglobulin superfamily. CD147 plays a regulatory role in the development of coronary artery disease by influencing plaque stability, platelet activation, and inflammatory responses (10). Changes in the expression and function of CD147 on the surface of platelets and monocytes have been observed with alterations in the state of coronary artery disease (11), yet its specific role and expression differences between Stable Angina (SA) and ACS patients remain unclear.

This study employed high-throughput sequencing technology to analyze whole blood samples from SA and ACS patients, aiming to screen and identify differential expression patterns of the CD147 gene to further understand its role in cardiovascular disease. Additionally, we utilized flow cytometry to quantitatively analyze the levels of CD147 on the surface of platelets and monocytes. This integrated approach not only provided expression data at the single-cell level but also revealed changes in CD147 expression across different states of coronary artery disease. Through this research, we hope to delve deeper into the biological role of CD147 in cardiovascular disease, offering new strategies for the prevention and treatment of coronary artery disease.

## Methods

### Study design and setting

**Figure 1:**
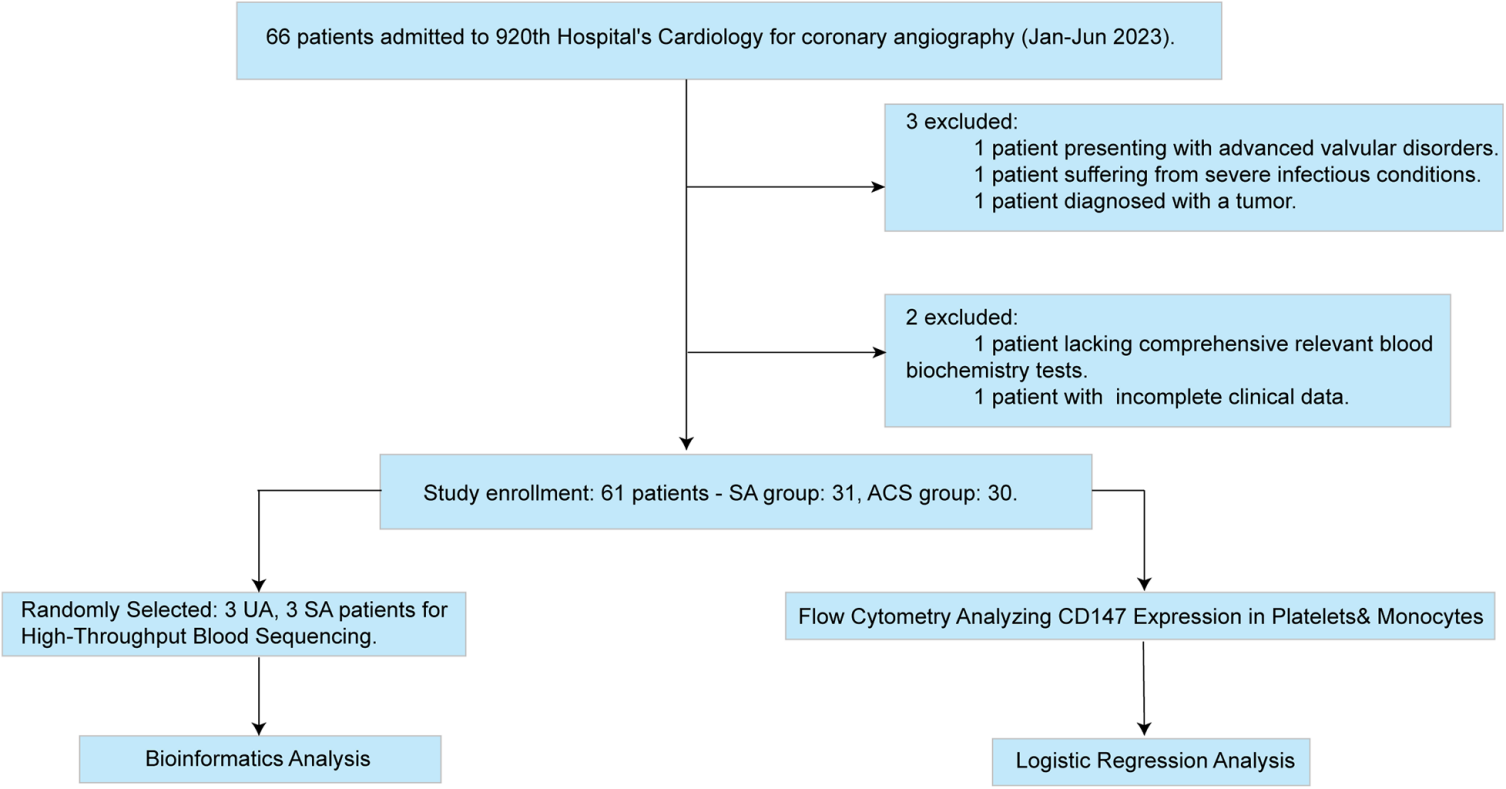
Study recruitment and analysis flowchart.

This investigation adopted a cross-sectional study design, involving 66 participants, all of whom were sourced from the Department of Cardiology at the 920th Hospital of Joint Logistics Support Force (Figure 1). These patients underwent coronary angiography between January and June 2023, with their ages ranging from 20 to 75 years.

### Inclusion and exclusion criteria

The inclusion criteria for this study are as follows: 1. Patients must have a diagnosis of coronary heart disease that conforms to the diagnostic standards of the American College of Cardiology/American Heart Association (ACC/AHA) (12), including 34 patients with Acute Coronary Syndrome (ACS) and 32 patients with Stable Angina (SA); 2. Patients must be able to actively cooperate with the completion of coronary angiography; 3. Patients diagnosed with coronary heart disease for the first time; 4. Patients must have complete clinical data; 5. Patients must be aged 20 years or above and below 75 years. The exclusion criteria include: (i) Patients with severe heart failure, cardiac valve disease, congenital heart disease, severe infection, severe liver or kidney dysfunction, or tumors; (ii) Patients with contraindications to antiplatelet or anticoagulant therapy (such as active peptic ulcer, thrombocytopenia, severe coagulation dysfunction, or hemorrhagic disease); (iii) Patients found to have coronary slow flow phenomenon during coronary angiography. These screening criteria were designed to ensure that the selected study subjects strictly meet the clinical state requirements of the research. After screening, 31 SA patients and 30 ACS patients were finally selected as the control group. Considering this study involves drawing venous blood from patients, participants were required to fully understand the study content and sign an informed consent form before participation. All experimental operations were strictly conducted in accordance with the Declaration of Helsinki of 2013 and had been approved by the Ethics Committee of our institution, ensuring that the study was conducted following ethical principles.

### Variables

#### Baseline characteristics

These include age, gender, height, weight, Body Mass Index (BMI), smoking history, history of hypertension, and diabetes.

#### Laboratory measurements

For all participants in the study, 5 milliliters of venous blood were collected within the first 4 h of admission [immediately for patients with Acute Coronary Syndrome (ACS)] to perform a comprehensive series of laboratory tests. These tests encompassed various domains, specifically: Hematologic analysis, which covers the percentage and absolute count of monocytes, platelet count, platelet distribution width, mean platelet volume, plateletcrit, and the proportion of large platelets. Blood glucose and metabolism-related indicators, including fasting blood glucose and uric acid levels. Cardiovascular disease-related biochemical markers such as Brain Natriuretic Peptide (BNP), Cardiac Troponin I (cTnI), Creatine Kinase (CK), and its isoenzyme (CK-MB). Renal function indicators, like serum creatinine (Cr) and cystatin C. Markers of inflammation and lipid metabolism, including C-reactive protein, triglycerides, total cholesterol, Low-Density Lipoprotein Cholesterol (LDL-C), High-Density Lipoprotein Cholesterol (HDL-C), total bilirubin, indirect bilirubin, direct bilirubin, and gamma-glutamyl transferase (γ-GT).

### Coronary artery diagnosis and IVUS analysis process

#### Coronary angiography procedure

In this study, coronary angiography was performed by experienced cardiovascular interventional physicians using the Seldinger technique via the radial artery. Following the placement of a 6F guiding catheter sheath, comprehensive multi-angle angiography of both the left and right coronary arteries was conducted to obtain detailed vascular imaging.

#### Analysis and assessment of angiography results

The analysis of these angiographic images was conducted by senior associate physicians with over ten years of experience. They utilized both visual assessment and software-assisted measurements to evaluate the degree of vascular stenosis. Coronary artery disease was diagnosed when the diameter stenosis of the coronary artery exceeded 50%. Moreover, the area with the most severe stenosis was selected as the target plaque for intravascular ultrasound (IVUS) examination.

#### Application of IVUS technology and plaque analysis

Following coronary angiography, the senior associate physicians also performed IVUS examinations to accurately determine the characteristics of the target lesion plaque. Under electrocardiographic monitoring, Virtual Histology Intravascular Ultrasound (VH-IVUS) technology was employed for image acquisition. The criteria for diagnosing vulnerable plaques included: plaque burden exceeding 40% in at least three consecutive frames, a necrotic core exceeding 10% that is in direct contact with the lumen, or the necrotic core area constituting more than 10% of the total plaque area with more than 30° of the circumference in contact with the lumen (13, 14). These criteria facilitated the differentiation between patients with stable angina (SA) having stable plaques and those with acute coronary syndrome (ACS) presenting vulnerable plaques.

### High-throughput RNA sequencing analysis

#### Sample selection and processing

In this study, three patients with Stable Angina (SA) and three with Unstable Angina (UA) were randomly selected for high-throughput RNA sequencing analysis of their whole blood samples. Ten milliliters of whole blood from each patient was collected in tubes containing EDTA and immediately supplemented with RNALater™ RNA stabilization reagent (Beyotime, China), to inhibit RNase activity and ensure RNA stability.

#### RNA extraction and quality assessment

RNA was extracted from the whole blood samples using the RNA/DNA Co-extraction Kit (Beyotime, China). The quality and purity of the extracted RNA samples were then assessed using an Agilent Bioanalyzer with an RNA picochip, ensuring their suitability for subsequent sequencing steps. This step is crucial for the accuracy of the sequencing results.

#### Library preparation and sequencing

Subsequently, high-quality RNA was used to construct sequencing libraries using Illumina's TruSeq RNA Sample Preparation Kit (Illumina, USA). This step prepared the RNA samples for Next-Generation Sequencing requirements. The final sequencing was performed on the Illumina NextSeq 500 platform, with quality control and preliminary analysis of the raw sequencing data conducted using software tools provided by Illumina.

### Bioinformatics analysis

#### Differential expression genes analysis

Differential expressed genes (DEGs) were identified using DESeq2 (version 1.42.0) in R version 4.2.0, with selection criteria set at *P* < 0.05 and |log2FC|≥0.87. All identified DEGs were utilized for subsequent analyses.

#### KEGG and GO enrichment analysis of DEGs

To better understand the biological functions and pathways of the DEGs, we performed functional enrichment analysis using the clusterProfiler package (15). clusterProfiler is a comprehensive tool that integrates information from over 40 independent sources, including GO and KEGG databases. Functional enrichment analysis of DEGs was conducted with parameters set to a minimum overlap of 2, a minimum enrichment factor of 1.5, and a significance threshold of *P* < 0.05.

#### Protein-protein interaction network construction

GeneMANIA (http://genemania.org) is a web tool designed for building functional association networks between genes and proteins (15). It predicts associations between genes by integrating various types of data from public databases, such as protein-protein interactions, co-expression, co-localization, and genetic interactions documented in the literature. In this study, the GeneMANIA database was used to analyze protein-protein interactions involving BSG and to explore functions and co-expressed proteins associated with BSG.

### Flow cytometry analysis

#### Venous blood collection

All included patients had their venous blood collected within 4 h of admission, with a volume of 10 ml collected. For patients with Acute Coronary Syndrome (ACS), the blood samples were collected immediately upon admission.

#### Platelet isolation

The collected blood was placed in anticoagulation tubes containing sodium citrate in a 1:9 volume ratio and fixed with 1% paraformaldehyde. Subsequently, it was centrifuged at 300 g for 10 min at room temperature to separate the plasma. The platelet-rich portion of the supernatant was transferred into 3 ml centrifuge tubes and diluted with Phosphate-Buffered Saline (PBS) before centrifugation to collect the platelet pellet at the bottom. This washing step was repeated three times to obtain pure platelets for analysis. The purified platelet samples were then dual-stained with FITC-conjugated CD147 antibody (eBioscience, USA) and PE-conjugated CD61 antibody (eBioscience, USA).

#### Monocyte isolation

Fresh anticoagulated blood was diluted with an equal volume of PBS. A suitable volume of separation medium was added into centrifuge tubes, and the diluted blood was layered gently on top of the separation medium, ensuring a clear interface between the two liquids. After centrifugation at 900 g for 20 min, the mononuclear cell layer located at the interface between the plasma and separation medium was collected. The cells were washed in PBS, centrifuged for 10 min, and the supernatant was discarded. This process was repeated twice, and the mononuclear cells were resuspended in PBS for the final pellet. The obtained mononuclear cell samples were subsequently dual-stained with FITC-conjugated CD147 antibody (eBioscience, USA) and PE-conjugated CD14 antibody (eBioscience, USA).

#### Flow cytometric analysis

CD147 expression levels were determined using an EPICS XL flow cytometer (Beckman Coulter, USA). CD147 expression levels were quantified by comparing with standard control samples and measured as Relative Fluorescence Intensity (RFI).

### Standards of measurement for variables

#### Baseline data

Data collection on patients’ medical histories is conducted by licensed physicians, strictly following diagnostic guidelines for determining past medical conditions, including:

#### Type 2 diabetes

Diagnosed according to the 《Guidelines for the Prevention and Treatment of Type 2 Diabetes in China》, which includes presenting typical symptoms of diabetes (excessive thirst, frequent urination, increased appetite, unexplained weight loss) and having a random venous plasma glucose ≥11.1 mmol/L, fasting venous plasma glucose ≥7.0 mmol/L, or a 2-hour plasma glucose ≥11.1 mmol/L after an oral glucose tolerance test (OGTT).

#### Hypertension

Diagnosed according to the 《Chinese Guidelines for the Prevention and Treatment of Hypertension》, defined by measurements taken on three different occasions (without the use of antihypertensive medication) showing a systolic blood pressure ≥140 mmHg and/or diastolic blood pressure ≥90 mmHg.

#### Smoking history

Individuals who have smoked continuously or cumulatively for 6 months or more in their lifetime or have a lifetime cumulative total of over 100 cigarettes.

#### BMI calculation

The formula for calculating Body Mass Index (BMI) is weight (kg)/[height (m)]^2^.

#### Laboratory biochemical indicators

All participants’ blood biochemical indicators are tested in the laboratory department of the 920 Hospital.

### Bias

In this study, particular emphasis is placed on ensuring the accuracy of case diagnoses and patients’ medical history to avoid biases caused by diagnostic errors. To minimize information bias, especially in studies related to CD147, all instruments and equipment used in the experiments underwent rigorous quality review and calibration processes. To reduce bias resulting from misclassification, this study employed Intravascular Ultrasound (IVUS) technology for precise identification of plaque stability. This step is crucial for enhancing the accuracy of plaque classification.

### Sample size

In conducting logistic regression multivariate analysis, a common empirical rule is that the sample size should be at least 5 times, 10 times, or ideally 20 times the number of variables included. Adopting a 5 times sample size is often considered the minimum acceptable standard, while following the 20 times rule is seen as the most stringent requirement (16). Based on the aforementioned rule, the calculated minimum sample size requirement is 45. Our study includes 61 samples, which exceeds the minimum sample size requirement calculated based on the number of variables needed, ensuring the effectiveness and reliability of the analysis.

### Statistical methods

Quantitative data conforming to a normal distribution are presented as mean ± standard deviation (x¯ ± s), and intergroup comparisons are made using the *t*-test. Quantitative data not following a normal distribution are represented using the quartile method and analyzed using the Mann-Whitney *U* test. Categorical data are presented as [*n* (%)], with the Chi-square test applied for comparisons. When the expected frequency is <5, the corrected Chi-square test is utilized. Indicators with statistical significance in univariate analysis are included in a binary multivariate Logistic regression analysis to evaluate the diagnostic value of CD147 on the surface of monocytes and platelets for ACS. A *P*-value <0.05 is considered statistically significant. All statistical analyses are performed using R software (version 4.2.0).

## Results

### Bioinformatics analysis

#### Identification of differential expressed genes (DEGs)

**Figure 2:**
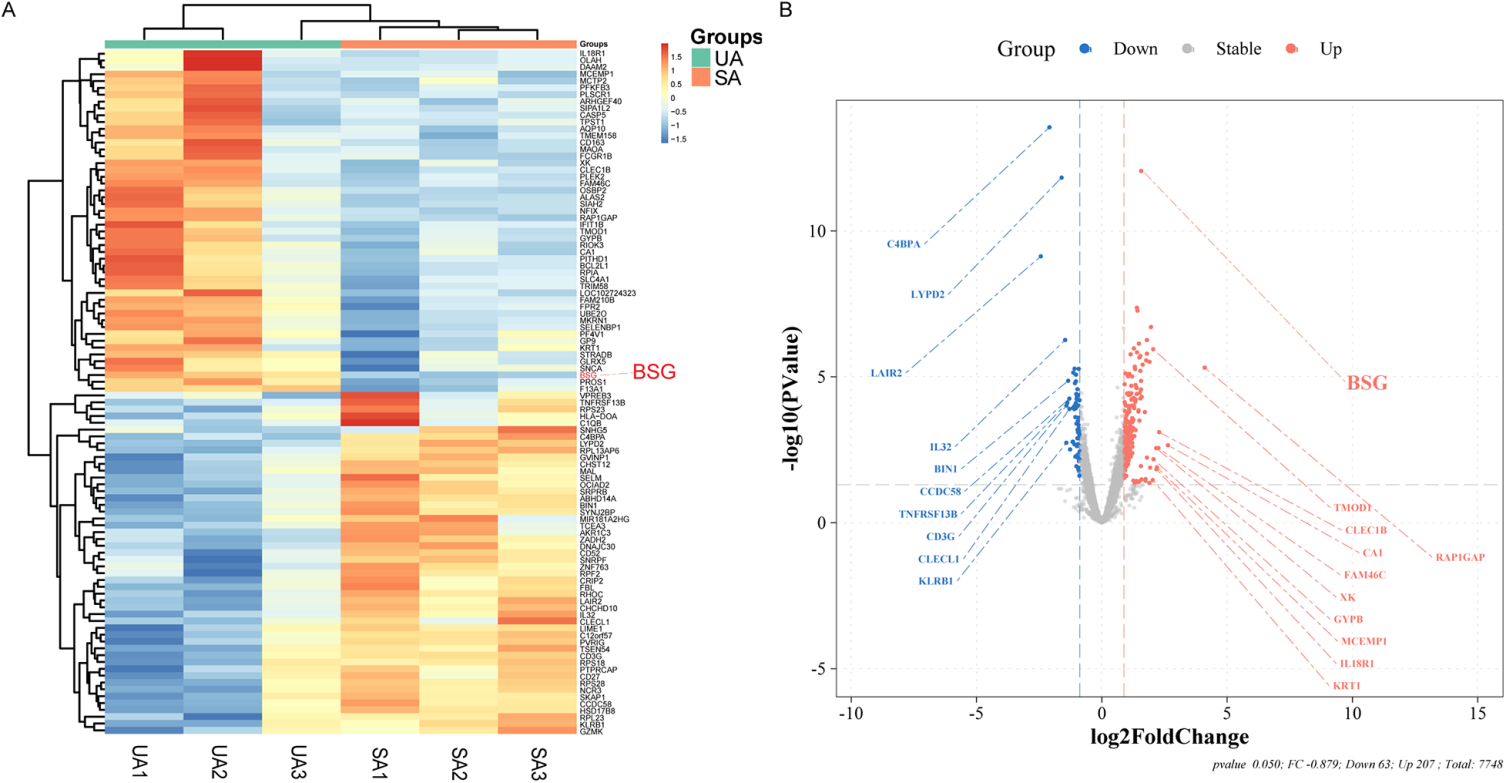
Differential Gene Expression Analysis from Stable Angina (SA) and Unstable Angina (UA) Patients. **(A)** Heatmap illustrating the differential expression of genes between patients with unstable angina (UA) and stable angina (SA). The color gradient represents the relative expression levels, with red indicating upregulation and blue indicating downregulation. BSG (CD147) is highlighted in red. **(B)** Volcano plot showing the log2 fold change of differentially expressed genes between UA and SA patients. Genes that are significantly upregulated in UA are shown in red, while those downregulated in UA are shown in blue. BSG (CD147) is highlighted in red on the plot.

In this study, utilizing high-throughput sequencing technology, we successfully identified 270 DEGs, of which 207 were upregulated and 63 were downregulated. In ACS, the expression levels of these DEGs changed by a factor of 1.8 or more compared to SA (detailed in Figure 2 and Supplementary Table S1).

#### Gene function and pathway enrichment analysis

**Figure 3:**
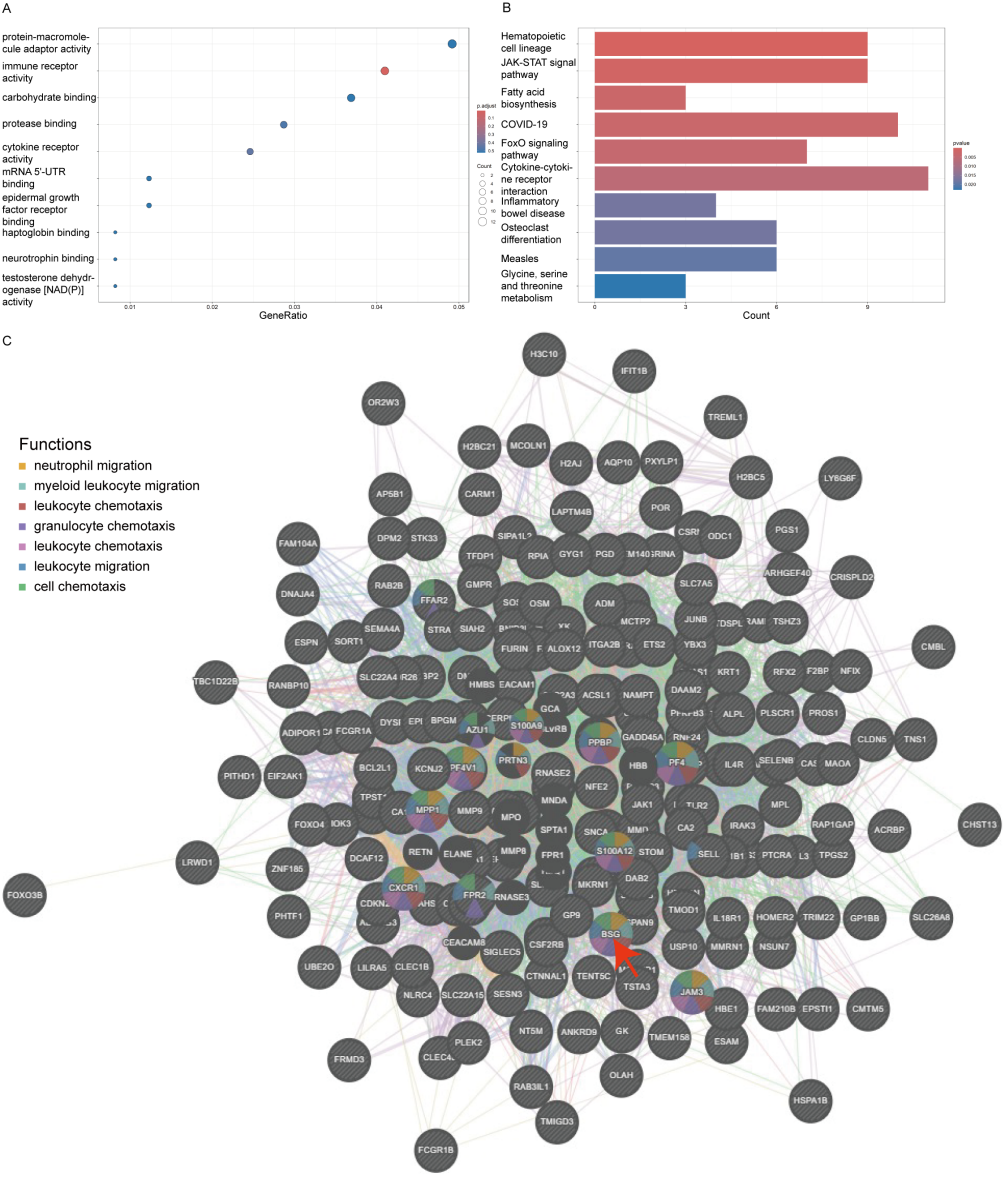
Go and KEGG enrichment of DEGs and PPI network. Panel **(A)** shows a bubble plot for Gene Ontology (GO) terms, where the size of the bubble corresponds to the gene count and the color intensity reflects the *P*-value, emphasizing the most significant GO terms like immune receptor and cytokine receptor activity. Panel **(B)** is a bar graph depicting the Kyoto Encyclopedia of Genes and Genomes (KEGG) pathway enrichment, with the length of the bars representing the gene count involved in each pathway and the color denoting the *P*-value. Panel **(C)** depicts a protein-protein interaction network with BSG (CD147) at the focal point, interacting with clusters of other proteins. The nodes are color-coded to indicate the functions BSG is associated with, such as leukocyte migration and chemotaxis, illustrating its vital role in these biological processes within the network.

Using the clusterProfiler package, we conducted a comprehensive Gene Ontology (GO) annotation and Kyoto Encyclopedia of Genes and Genomes (KEGG) pathway enrichment analysis of these DEGs, considering all genes within the genome. By grouping genes to assess their similarity, we found that under the conditions of a *P*-value less than 0.05, an enrichment score over 1.5, and containing at least two genes, the top five GO enriched pathways ranked by -log*P* value included immune receptor activity, cytokine receptor activity, protease binding, mRNA 5'-UTR binding, and haptoglobin binding (Figure 3A). The top five KEGG enriched pathways were Hematopoietic cell lineage, JAK-STAT signaling pathway, Fatty acid biosynthesis, Coronavirus disease - COVID-19, and FoxO signaling pathway (Figure 3B).

#### PPI network

In this study, we utilized Genemania version 3.6.0 to analyze the physical protein-protein interactions (PPIs) among the 270 DEGs. In the constructed PPI network, CD147/BSG emerged as a key node, participating with other DEGs in various processes related to the chemotaxis and migration of white cells and granulocytes, specifically including neutrophil migration, myeloid leukocyte migration, leukocyte chemotaxis, granulocyte chemotaxis, leukocyte chemotaxis, leukocyte migration, and cell chemotaxis (Figure 3C).

### Particpants

In this study, a total of 61 patients were recruited, including 31 patients diagnosed with Stable Angina (SA) and 30 patients with Acute Coronary Syndrome (ACS). All participants strictly adhered to the predefined inclusion and exclusion criteria (Table 1).

**Table 1:** Characteristics of stable angina and acute coronary syndrome.

	SA	ACS	OR	*P*
	*N* = 31	*N* = 30		
Sex				0.308
Female	12 (38.7%)	7 (23.3%)	Ref.	
Male	19 (61.3%)	23 (76.7%)	2.04 [0.67; 6.58]	
Age (year)	68.0 [61.0; 72.5]	64.0 [54.0;69.5]	0.96 [0.91; 1.01]	0.122
BMI (kg/m^2^)	24.3 (3.45)	23.9 (3.32)	0.96 [0.83; 1.12]	0.627
Hypertension				0.146
No	7 (22.6%)	13 (43.3%)	Ref.	
Yes	24 (77.4%)	17 (56.7%)	0.39 [0.12; 1.17]	
Diabetes				1.000
No	25 (80.6%)	24 (80.0%)	Ref.	
Yes	6 (19.4%)	6 (20.0%)	1.04 [0.28; 3.87]	
Smoking history				1.000
No	15 (48.4%)	14 (46.7%)	Ref.	
Yes	16 (51.6%)	16 (53.3%)	1.07 [0.39; 2.98]	
WBC (*10^9^/L)	6.28 [5.44; 7.54]	11.8 [10.2; 13.2]	1.57 [1.25; 1.97]	<0.001
Mono.per (%)	5.95 (2.09)	4.23 (2.20)	0.68 [0.52; 0.90]	0.003
Mono (*10^9^/L)	0.35 [0.30; 0.44]	0.36 [0.27; 0.60]	1.54 [0.29; 8.28]	0.925
Platelet (*10^9^/L)	207 (51.5)	211 (30.5)	1.00 [0.99; 1.01]	0.714
PDW (fl)	13.1 [11.8; 16.0]	16.0 [12.3; 16.4]	1.14 [0.94; 1.39]	0.111
PCT (ng/ml)	0.21 (0.04)	0.21 (0.03)	0.16 [0.00; 108,757]	0.790
BNP (pg/ml)	40.4 [23.2; 66.8]	126 [37.6; 350]	1.01 [1.00; 1.01]	0.001
cTn (ng/L)	5.20 [2.15; 18.2]	17,654 [8,705; 83,448]	1.00 [1.00; 1.00]	<0.001
CRP (mg/L)	1.21 [0.90; 3.71]	2.50 [1.31; 20.2]	1.06 [1.00; 1.12]	0.028
LDL (mmol/L)	2.54 (0.96)	2.71 (0.74)	1.26 [0.69; 2.30]	0.450
CD147_Platelet	0.77 (0.28)	1.10 (0.19)	336 [15.8; 7,138]	<0.001
CD147_Mono	0.95 [0.91; 1.07]	0.96 [0.92; 1.05]	0.59 [0.03; 13.5]	0.778

Continuous data conforming to a normal distribution were reported as mean (SD) and those not conforming as median (quartiles). Statistical significance was determined using *t*-test, Rank-sum test or *χ*^2^ test, with *p*-values calculated.

SA, stable angina; ACS, Acute Coronary Syndrome; BMI, body mass index; WBC, white blood cell; Mono.Per, monocytes percentage; Mono, monocytes; PDW, platelet distribution width; MPV, mean platelet volume; PCT, procalcitonin; *P*-LCR, platelet -larger cell ratio; cTn, cardiac troponin; CRP, C-reactive protein; LDL, low-density lipoprotein.

### Descriptive data

#### Indicators with significant differences

In our study, statistical analysis revealed significant differences (*P* < 0.05) in the following indicators: total white blood cell count, percentage of neutrophils, percentage of monocytes, fasting blood glucose levels, cardiac disease marker BNP (Brain Natriuretic Peptide), myocardial-specific protein troponin, levels of creatine kinase and its isoenzymes, inflammatory marker C-reactive protein, lipid marker triglycerides, and liver function marker total bilirubin (detailed in Supplementary Table S1).

#### Indicators without significant differences

The analysis showed no statistically significant differences (*P* > 0.05) regarding gender, age, history of hypertension, Body Mass Index (BMI), smoking habits, total monocyte count, total platelet count, and the infection marker procalcitonin.

### Flow cytometry analysis

The flow cytometry results, as detailed in (Table 1, Supplementary Figures S1–S4), provide descriptive statistics for the expression of CD147 on the surface of platelets. In the Stable Angina (SA) group, the mean expression level of CD147 on platelets was 0.77 ± 0.28, whereas in the Acute Coronary Syndrome (ACS) group, it was 1.10 ± 0.19. The difference between the two groups was statistically significant, with a *P*-value of <0.001. For monocyte surface CD147 expression, the SA group had a median value of 0.95 [interquartile range (IQR): 0.91, 0.97], compared to the ACS group, which had a median of 0.96 [IQR: 0.92, 1.05]. The difference between the groups did not reach statistical significance (*P* = 0.778).

### Logistic regression analysis

**Figure 4:**
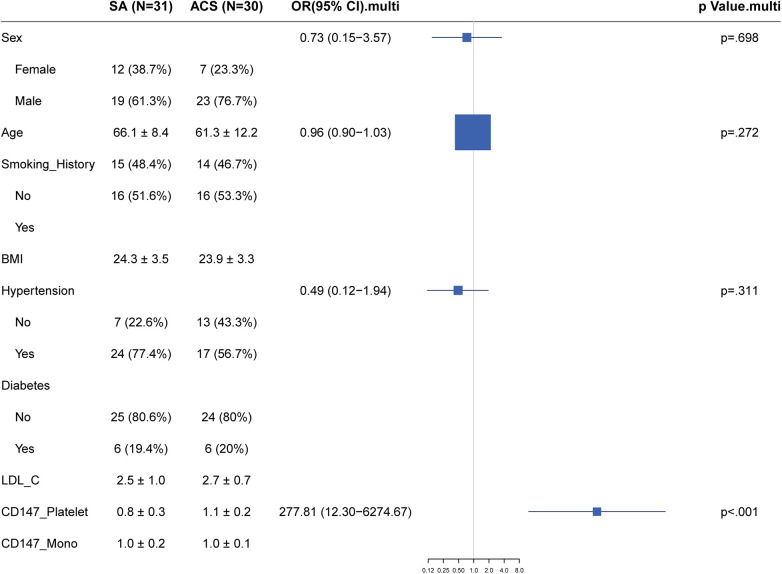
Forest plot of traditional risk factor odds ratios for stable angina vs. Acute Coronary Syndrome with emphasis on CD147 expression.

Using a logistic regression model, we assessed the relationship between the expression of CD147 on the surface of platelets and the presence of Stable Angina (SA) vs. Acute Coronary Syndrome (ACS). After controlling for traditional coronary artery disease risk factors such as gender, age, Body Mass Index (BMI), smoking history, hypertension, diabetes, and Low-Density Lipoprotein Cholesterol (LDL-C), we found that the level of CD147 expression on platelets was significantly positively correlated with the risk of Acute Coronary Syndrome (ACS). The adjusted odds ratio was 277.81, with a 95% confidence interval ranging from 12.3 to 6,274.67, and a *P*-value of <0.001 (Figure 4). However, in this model, the effects of gender, BMI, hypertension, diabetes, and LDL-C on predicting the risk of ACS did not reach statistical significance.

## Discussion

This study explored the significance and impact of CD147 expression differences between patients with Stable Angina Pectoris (SAP) and Acute Coronary Syndrome (ACS). Through high-throughput sequencing of whole blood samples, we identified 270 differentially expressed genes (DEGs), with CD147 serving as a key node, indicating its potential pivotal role in the stability of atherosclerotic plaques. Flow cytometry analysis of CD147 expression on platelets and monocytes revealed that the expression level of CD147 on platelets was significantly higher in the ACS group compared to the SAP group (1.10 ± 0.19 vs. 0.77 ± 0.28, *P* < 0.001), while the difference in CD147 expression on monocytes between the two groups was not statistically significant (0.96 [0.92, 1.05] vs. 0.95[0.91, 0.97]). Multivariate logistic regression analysis demonstrated a significant association between the expression of CD147 on platelets and plaque stability (adjusted odds ratio = 277.81, 95% confidence interval: 12.3–6,274.67, *P* < 0.001). These results may suggest that the expression level of CD147 on platelets is not only associated with plaque stability but could also indicate a causal relationship.

CD147 is involved in several critical biological processes, including immune response, inflammation, and cellular adhesion (17). One of the primary mechanisms by which CD147 may influence plaque stability is through its role in the regulation of matrix metalloproteinases (MMPs) (18). MMPs are enzymes responsible for degrading the extracellular matrix, which can lead to the thinning of the fibrous cap of atherosclerotic plaques, making them more prone to rupture (19). The upregulation of CD147 on the surface of platelets and other cells within the plaque environment could enhance MMP activity, thereby contributing to plaque instability (18, 20). Additionally, CD147 has been shown to interact with various signaling pathways, including the JAK-STAT pathway, which is crucial for cytokine production and immune cell proliferation (21). This interaction suggests that CD147 might play a role in promoting a pro-inflammatory environment within the plaque, further exacerbating plaque vulnerability (22). Moreover, recent studies suggest that CD147 may influence platelet activation and aggregation, processes that are directly involved in thrombus formation following plaque rupture (23). The elevated expression of CD147 observed in patients with unstable angina could, therefore, be a contributing factor to the increased risk of acute coronary events in these patients (24).

The lack of DNA in platelets means that differences in the protein expression of CD147 on their surface could be attributed to several factors:1. Differential expression of CD147 in maturing megakaryocytes in the bone marrow could be induced by certain stimuli (25). These precursors of platelets could exhibit variation in CD147 expression due to environmental or physiological cues, leading to a heterogeneous population of platelets with varying levels of CD147 once they enter the circulation. 2. An increase in the translation of CD147 in circulating platelets could be triggered by certain stimuli. Since platelets can synthesize proteins from pre-existing mRNA, factors such as inflammatory cytokines or cellular interactions within the bloodstream might enhance the translation of CD147 mRNA (26), contributing to the elevated protein levels observed. 3. In the case of the SA group, a decrease in platelet CD147 expression might be influenced at the DNA level in the precursor cells, such as megakaryocytes (27). High methylation of the CD147 gene in SA could inhibit its expression, leading to lower levels of CD147 protein on the surface of platelets derived from these cells. Methylation is a common epigenetic modification that can suppress gene expression, and its involvement would suggest a more systemic regulatory mechanism affecting platelet function and characteristics in stable angina conditions (28).

Our study identifies a significant upregulation of CD147 on platelets in ACS patients, which is consistent with previous findings that associate CD147 with plaque instability. For example, Huang et al. demonstrated that CD147 correlates with MMP-9 activity, leading to extracellular matrix degradation and increased plaque vulnerability (20). In contrast to studies focusing on CD147 in monocytes and endothelial cells, our research uniquely highlights platelet CD147 expression as a critical marker for cardiovascular pathology, distinguishing between SA and ACS. This focus provides new insights into the specific role of platelets in plaque instability, which has been underexplored in existing literature (18). Furthermore, our logistic regression analysis reveals a strong association between platelet CD147 levels and the risk of acute coronary syndrome (ACS), suggesting its potential as a prognostic marker. This aspect of CD147's role in cardiovascular disease expands on previous studies, which have primarily examined its involvement in other pathological conditions such as cancer and inflammation (24). By incorporating these comparisons, our study not only aligns with but also extends the current understanding of CD147's function in cardiovascular diseases, particularly in the context of plaque stability and ACS risk.

Future research should focus on how these regulatory mechanisms affect CD147 expression and its impact on plaque stability. Specifically, this includes: 1. Further clarification of the causal links between CD147 and enriched pathways, as well as their impact on plaque stability, can be achieved through studies on cellular and animal models. This approach would allow for a deeper understanding of the mechanistic roles CD147 plays in cardiovascular pathology. 2. Regarding the differential expression of CD147 on platelets, existing evidence indicates a close association of CD147 with hyaluronic acid (17, 24), gene polymorphisms (29), and methylation regulation (30). There is an opportunity to examine the expression of CD147 in megakaryocytes within the bone marrow through bone marrow biopsy in clinical samples. This could provide insights into the transcriptional and epigenetic mechanisms influencing CD147 expression before platelet formation. 3. Future research should consider conducting longitudinal studies to track changes in CD147 expression over time in patients with cardiovascular diseases. Such studies would be valuable in understanding the dynamic nature of CD147 expression in relation to disease progression or response to therapy. Ultimately, clinical trials could evaluate the effectiveness of therapeutic interventions targeting CD147, offering new directions for the treatment of cardiovascular diseases.

However, our study also has certain limitations. The relatively small sample size and the predominance of male participants may limit the generalizability of our findings, particularly concerning sex differences in CD147 expression and cardiovascular disease (CVD) progression. Additionally, as a cross-sectional study, it does not allow for direct inference of causality. Further research is required to validate our findings and explore the exact relationship between changes in CD147 expression and the development of cardiovascular diseases. Moreover, the expression of CD147 on the surface of platelets, with a wide 95% confidence interval, suggests considerable variability that warrants closer examination in larger, more diverse cohorts. Future studies might also benefit from a longitudinal design to better understand the temporal relationship between CD147 expression and cardiovascular disease progression.

## Conclusion

Our study highlights the potential value of CD147 in distinguishing between Stable Angina Pectoris and Acute Coronary Syndrome, offering new insights for the diagnosis and treatment of cardiovascular diseases. Despite its limitations, our findings lay an important foundation for future research, especially in exploring the potential of CD147 as a therapeutic target for cardiovascular diseases. As our understanding of the complex mechanisms of this disease deepens, research on CD147 will continue to unveil new pathways for the treatment and prevention of cardiovascular diseases.

## Data Availability

The datasets generated and/or analyzed during the current study have been deposited in the Sequence Read Archive (SRA) and are publicly available. The data can be accessed at the following link: https://www.ncbi.nlm.nih.gov/sra/PRJNA1154818 with the accession number PRJNA1154818. For any additional information or specific data requests, please contact the corresponding author.
