# Supplementary material for: Integrating genomic profiling to clinical data: assessing the impact of CD147 expression on plaque stability
Source: Front Cardiovasc Med. 2024 Sep 13;11:1425817. doi: 10.3389/fcvm.2024.1425817 (PMC11444025; doi:10.3389/fcvm.2024.1425817)
Supplement: Supplementary file 1 [file Datasheet1.pdf]

TableS 1. Differential Expressed Genes between Stable Angina (SA) and Unstable Angina by High-Throughput Sequencing.

Symbol	baseMean	log2FoldChange	lfcSE	stat	pvalue	padj	group
C4BPA	267.2676136	-2.091618366	0.27497661	-7.606531945	2.82E-14	2.18E-10	Down
BSG	540.1152909	1.569475976	0.219568897	7.147988617	8.81E-13	3.41E-09	Up
LYPD2	178.6117781	-1.606004365	0.226997117	-7.074998946	1.49E-12	3.86E-09	Down
LAIR2	605.9137371	-2.437138409	0.395840156	-6.156875123	7.42E-10	1.44E-06	Down
PROS1	255.4979362	1.397726821	0.255142623	5.478217642	4.30E-08	6.66E-05	Up
F13A1	587.3445266	1.419653459	0.261076391	5.437693741	5.40E-08	6.97E-05	Up
SELENBP1	2620.824968	1.960137222	0.376752177	5.202723014	1.96E-07	0.000208514	Up
IL32	892.4199211	-1.464787519	0.292422152	-5.009153746	5.47E-07	0.000425955	Down
OSBP2	685.2064448	1.795444534	0.358509663	5.008078491	5.50E-07	0.000425955	Up
UBE2O	427.7675271	1.477797513	0.298346168	4.953298114	7.30E-07	0.000513946	Up
CA2	235.5876787	1.277325131	0.261777033	4.879439249	1.06E-06	0.000675736	Up
TMOD1	788.9947796	2.050703316	0.421359624	4.866871909	1.13E-06	0.000675736	Up
MKRN1	16815.01683	1.401665265	0.290925123	4.81795883	1.45E-06	0.000802661	Up
SIAH2	790.7787445	1.797531128	0.374676334	4.797557162	1.61E-06	0.00081985	Up
YBX3	15680.47481	1.147850518	0.239785553	4.786987812	1.69E-06	0.00081985	Up
FPR2	1133.203368	1.536885234	0.322779582	4.761407846	1.92E-06	0.000876194	Up
FAM210B	1103.797402	1.498942248	0.31640369	4.737436052	2.16E-06	0.00093165	Up
RIOK3	1463.191089	1.768890109	0.377217034	4.689316624	2.74E-06	0.001117828	Up
TMEM158	868.428577	1.902331313	0.40775501	4.665378147	3.08E-06	0.001193386	Up

TRIM58	1070.644785	1.629282144	0.352718318	4.619216135	3.85E-06	0.001421177	Up
ACRBP	862.398553	1.313326245	0.286384459	4.585885172	4.52E-06	0.001592098	Up
ITGB3	195.9301725	1.326698738	0.291749423	4.547391135	5.43E-06	0.001618587	Up
PAQR8	421.4028434	-0.922608482	0.202713873	-4.551284367	5.33E-06	0.001618587	Down
RAP1GAP	979.6529158	4.10338307	0.897536119	4.571830575	4.83E-06	0.001618587	Up
RPL13AP6	4613.260465	-1.088172156	0.238949377	-4.553986156	5.26E-06	0.001618587	Down
NFIX	299.4593256	1.535991398	0.339888067	4.519109516	6.21E-06	0.001782048	Up
CD52	11893.06133	-1.14141958	0.254298247	-4.488507456	7.17E-06	0.001984703	Down
FAM104A	2832.112951	0.945311092	0.211240658	4.475043296	7.64E-06	0.002041086	Up
CSF2RB	161.817068	1.098330767	0.245896754	4.466633857	7.95E-06	0.002052186	Up
ZADH2	300.232755	-1.035870259	0.232624211	-4.452976993	8.47E-06	0.002116649	Down
HSPA1B	691.4286688	1.209166671	0.273377488	4.423065992	9.73E-06	0.002284721	Up
RNF123	363.3497481	1.047251698	0.236620299	4.425874289	9.61E-06	0.002284721	Up
BIN1	1680.40514	-1.343226793	0.308777051	-4.350150988	1.36E-05	0.003046828	Down
STRADB	9744.737039	1.562973333	0.359502412	4.347601796	1.38E-05	0.003046828	Up
RHOC	642.5189805	-1.036464291	0.238864021	-4.339139428	1.43E-05	0.003078576	Down
OCIAD2	399.6723565	-1.073919633	0.249328636	-4.30724544	1.65E-05	0.003461475	Down
ZNF185	498.5193423	0.910126668	0.212231276	4.288372039	1.80E-05	0.003669849	Up
LOC102724323	185.3067311	1.344197385	0.314438247	4.274916928	1.91E-05	0.003798677	Up
TNS1	1047.733686	1.173815674	0.275475894	4.261046794	2.03E-05	0.003941245	Up
ALAS2	2029.302355	1.373165584	0.324601954	4.230305976	2.33E-05	0.004315667	Up

CD163	424.332839	1.390406842	0.329306821	4.222223018	2.42E-05	0.004358783	Up
ABHD14A	337.5322406	-0.977808145	0.232758064	-4.200963565	2.66E-05	0.004666939	Down
PLEK2	404.1131041	1.562301078	0.372285356	4.196514993	2.71E-05	0.004666939	Up
IGF2BP2	1001.284308	1.175733198	0.284812607	4.128093938	3.66E-05	0.005771473	Up
LYZ	3190.282357	1.007766531	0.244369954	4.12393797	3.72E-05	0.005771473	Up
PLSCR1	226.9789856	1.509090205	0.365016943	4.134301797	3.56E-05	0.005771473	Up
STOM	626.9392522	1.066616664	0.258103422	4.132516557	3.59E-05	0.005771473	Up
RPIA	793.1700094	1.387328454	0.337288686	4.113178158	3.90E-05	0.00581471	Up
SRPRB	789.9994297	-1.018463829	0.247508072	-4.114871172	3.87E-05	0.00581471	Down
DNAJC30	486.4902727	-0.949304208	0.231721149	-4.096752552	4.19E-05	0.005906993	Down
MCOLN1	1409.558251	1.066707809	0.260390385	4.096571417	4.19E-05	0.005906993	Up
NAMPT	1771.722925	1.122843383	0.273710441	4.102303809	4.09E-05	0.005906993	Up
CHST12	284.5689851	-0.990970104	0.244056398	-4.060414362	4.90E-05	0.006658618	Down
FCGR1B	1067.44912	1.590265143	0.391457078	4.06242531	4.86E-05	0.006658618	Up
GP1BB	13789.29366	1.26646049	0.313395053	4.041099179	5.32E-05	0.006986496	Up
BCL2L1	2544.864234	1.357865276	0.338405249	4.012542008	6.01E-05	0.007062985	Up
CCDC58	167.1571909	-1.297673451	0.322048896	-4.029429894	5.59E-05	0.007062985	Down
CHCHD10	1710.864496	-1.004679704	0.249809062	-4.021790469	5.78E-05	0.007062985	Down
MPL	207.2358927	1.17925337	0.294064739	4.010182835	6.07E-05	0.007062985	Up
MPP1	3160.279149	1.18565342	0.29510722	4.017703869	5.88E-05	0.007062985	Up
OR2W3	629.3946731	1.277211761	0.318320692	4.012342879	6.01E-05	0.007062985	Up

SLC4A1	1593.621574	1.540779595	0.382011836	4.033329462	5.50E-05	0.007062985	Up
THEM6	506.5099642	-0.896152434	0.224148953	-3.998021947	6.39E-05	0.007277882	Down
GLRX5	10876.94375	1.333381019	0.335678935	3.972191517	7.12E-05	0.007996658	Up
EPB42	3761.643012	0.909698933	0.229551692	3.96293718	7.40E-05	0.008194425	Up
TNFRSF13B	227.2823782	-1.374974644	0.347579951	-3.955851424	7.63E-05	0.008322285	Down
SELM	173.8721986	-1.041442008	0.263980454	-3.945148177	7.98E-05	0.008582056	Down
SLC25A37	19957.28361	0.930774583	0.237331495	3.921833402	8.79E-05	0.009327079	Up
CD3G	533.2825765	-1.410499286	0.361798551	-3.898576379	9.68E-05	0.009782261	Down
EIF2AK1	4523.15103	0.963967555	0.247463801	3.895388132	9.80E-05	0.009782261	Up
HBE1	788.8597845	1.078188021	0.277082299	3.891219408	9.97E-05	0.009782261	Up
SYNJ2BP	569.7041035	-0.935975495	0.240055283	-3.898999776	9.66E-05	0.009782261	Down
TGFA	216.5988255	1.211654127	0.310760493	3.898996667	9.66E-05	0.009782261	Up
TREML1	278.2470682	1.277753598	0.328161792	3.893669615	9.87E-05	0.009782261	Up
AKR1C3	528.22012	-1.099513527	0.282794116	-3.888035376	0.000101059	0.009787554	Down
C12orf57	1889.733116	-1.143270457	0.296538433	-3.855387126	0.000115547	0.010786209	Down
ESAM	238.8040656	1.290803479	0.335321434	3.849451151	0.000118383	0.010790939	Up
GVINP1	3018.652888	-1.035763841	0.269029528	-3.850000586	0.000118118	0.010790939	Down
CLECL1	157.1913296	-1.291580237	0.336849999	-3.834288971	0.000125928	0.011214832	Down
WDR26	338.9283186	1.08933258	0.28394838	3.836375399	0.000124864	0.011214832	Up
CRIP2	266.1399837	-1.099746056	0.28708175	-3.830776623	0.000127739	0.011246877	Down
PITHD1	4335.728468	1.467972617	0.385514014	3.807832047	0.00014019	0.012068838	Up

EPB41	134.9428746	0.925463986	0.243984729	3.793122586	0.000148765	0.012666249	Up
GP9	1977.127563	1.456831102	0.384994572	3.784030241	0.000154309	0.012914512	Up
SLC7A5	355.1218011	1.092915067	0.288949581	3.782372905	0.00015534	0.012914512	Up
SNCA	5159.508397	1.714671596	0.454601533	3.771812169	0.000162066	0.013217781	Up
HEMGN	130.6692082	1.03340028	0.275034927	3.75734199	0.000171728	0.013716974	Up
DMTN	1947.395244	0.991748594	0.266969261	3.714841884	0.000203331	0.015913202	Up
HSD17B8	233.256539	-1.057210909	0.287874979	-3.672465429	0.000240222	0.018247425	Down
ZNF763	165.1016483	-0.953545854	0.260481674	-3.660702266	0.000251525	0.018738605	Down
TSTA3	420.3792334	1.211217004	0.331700936	3.651533271	0.000260679	0.019235651	Up
TPGS2	581.5579434	0.954345674	0.261878947	3.644224498	0.000268199	0.019420633	Up
GADD45A	281.9539209	1.262644296	0.349062375	3.617245475	0.000297755	0.021165183	Up
PFKFB3	888.6253249	1.498152971	0.415931882	3.60191905	0.000315877	0.022057889	Up
RANBP10	419.2265609	0.981841246	0.272596509	3.601811522	0.000316007	0.022057889	Up
SNRPF	1364.457698	-0.958578044	0.267176432	-3.587809133	0.000333468	0.023068857	Down
FBXO7	1035.237532	1.222296665	0.3423659	3.570147217	0.000356781	0.023830487	Up
HIST2H2BE	538.1515395	1.160646708	0.325070703	3.570443899	0.000356377	0.023830487	Up
JAM3	378.614098	1.049515612	0.293617524	3.574431116	0.00035099	0.023830487	Up
SDCBP	1912.917305	1.12793648	0.315477931	3.575326101	0.000349792	0.023830487	Up
RAB3IL1	629.5738602	1.154904868	0.32422635	3.562032723	0.000367995	0.024369414	Up
ALOX12	257.0606513	0.953166457	0.26829204	3.552719856	0.00038127	0.024824219	Up
FBL	1402.885897	-1.011308001	0.285818085	-3.538292553	0.000402724	0.02600252	Down

CARM1	3040.061145	0.928780218	0.263990104	3.518238771	0.000434421	0.027144324	Up
PVRIG	766.5684698	-0.927038918	0.264045461	-3.510906478	0.000446581	0.027680906	Down
TFDP1	705.1339835	1.119901994	0.319264189	3.507759504	0.000451897	0.027788102	Up
ANXA3	136.8728337	0.917592573	0.264749436	3.465890574	0.000528478	0.031500173	Up
GYG1	1856.78298	1.10323306	0.318313813	3.46586612	0.000528526	0.031500173	Up
CTNNAL1	136.5150096	1.045636496	0.302156435	3.460579931	0.000539013	0.031879956	Up
SLC22A4	535.4517604	1.199802074	0.347261491	3.455039229	0.000550213	0.032052996	Up
TMIGD3	158.2507065	1.205276608	0.348779359	3.45569936	0.000548867	0.032052996	Up
NSUN7	207.8684467	1.245243124	0.361178082	3.447726165	0.000565327	0.032687706	Up
ACSL1	1068.338559	1.126397237	0.326942311	3.445247674	0.000570536	0.032744561	Up
METTL5	387.3486116	-0.923412372	0.268288255	-3.441866552	0.000577715	0.032912773	Down
ALPL	11978.85293	1.241906322	0.364502121	3.407130583	0.000656497	0.035079583	Up
MAL	1023.727604	-0.970141378	0.284660063	-3.408069852	0.000654241	0.035079583	Down
SORT1	819.04551	1.309688059	0.383915393	3.411397623	0.000646308	0.035079583	Up
PF4V1	383.2273066	1.347438777	0.396070464	3.402017823	0.000668903	0.035497661	Up
GNAQ	222.496414	1.085226079	0.319662518	3.39491188	0.000686507	0.036184052	Up
ANKRD9	218.2057036	0.910030618	0.269340471	3.378737016	0.000728196	0.037560284	Up
C7orf50	1469.775192	-0.897531331	0.265848323	-3.376103035	0.000735204	0.037560284	Down
GK	959.2644069	1.185096309	0.350777184	3.378487434	0.000728858	0.037560284	Up
PTCRA	194.0235989	1.011888443	0.299453179	3.379120725	0.000727181	0.037560284	Up
ETS2	704.7772021	1.084574551	0.321656258	3.371843465	0.000746669	0.037811695	Up

CLEC1B	615.1563562	2.285729046	0.680732054	3.357751458	0.000785792	0.039068991	Up
DAB2	412.2741262	1.100261134	0.328332913	3.351053429	0.000805048	0.039068991	Up
RPF2	411.9687359	-0.949687878	0.283189485	-3.353542169	0.000797843	0.039068991	Down
TCEA3	189.7242665	-0.933565575	0.278638579	-3.350453402	0.000806794	0.039068991	Down
CRISPLD2	2467.931433	0.979125326	0.293727768	3.333444888	0.000857777	0.041025021	Up
TMEM140	4209.128467	1.037735008	0.312704568	3.318579623	0.000904765	0.042744641	Up
TOMM7	9536.920916	-0.893190766	0.269879487	-3.309591159	0.000934323	0.043476975	Down
LY6G6F	249.3629588	1.070901975	0.324076401	3.304473799	0.000951549	0.04388452	Up
LIN7A	245.8583219	1.138105493	0.345090771	3.297988787	0.0009738	0.044644995	Up
ADIPOR1	7367.109929	1.093970732	0.333812055	3.27720559	0.0010484	0.046417162	Up
HSPE1	446.9941243	-0.889526235	0.271426698	-3.277224535	0.00104833	0.046417162	Down
FRMD3	247.5108519	1.10279848	0.336916741	3.273207728	0.001063343	0.046811258	Up
ITGA2B	452.7565289	1.242409341	0.379949393	3.269933743	0.001075727	0.046824332	Up
RAB2B	2900.993067	1.298756461	0.39703736	3.271119026	0.001071228	0.046824332	Up
SOCS3	118.2353255	1.037466253	0.318542476	3.256916521	0.001126296	0.048480765	Up
CEACAM1	474.4894247	1.173025394	0.360773321	3.251419452	0.001148303	0.048884902	Up
RBM38	3487.22011	1.008542876	0.310520627	3.247909436	0.001162563	0.048983281	Up
FECH	143.7569115	0.890676987	0.274890647	3.240113827	0.00119482	0.049241834	Up
BCL3	762.5161974	1.002078482	0.311560448	3.216321222	0.001298454	0.04974828	Up
DNAJA4	842.7556104	1.202431673	0.373980497	3.215225617	0.00130342	0.04974828	Up
EPAS1	126.0718942	0.938624751	0.290318798	3.233082934	0.00122462	0.04974828	Up

FOXO4	812.7183916	1.203696666	0.374195147	3.216761829	0.001296462	0.04974828	Up
NCR3	438.2324463	-0.936168163	0.290973671	-3.217363826	0.001293744	0.04974828	Down
NFE2	12358.66853	0.892228346	0.275780768	3.235281247	0.00121523	0.04974828	Up
RPS28	1941.758617	-0.976848221	0.303104592	-3.222809051	0.001269401	0.04974828	Down
SOS1	126.0345757	1.058330793	0.329022835	3.216587672	0.001297249	0.04974828	Up
MMD	440.4234287	1.12577108	0.351566452	3.202157298	0.001364025	0.050809929	Up
SERPINB1	3181.658168	1.032370079	0.322383254	3.202306773	0.001363317	0.050809929	Up
SIGLEC5	645.8126893	1.027107507	0.321638447	3.193360483	0.001406273	0.051884767	Up
CYP1B1	343.8856015	0.89615638	0.281878664	3.179227433	0.001476682	0.053145424	Up
MICALCL	267.1785414	1.028085841	0.323473338	3.178270726	0.001481563	0.053145424	Up
TSHZ3	355.4693688	1.132381414	0.356289264	3.178264205	0.001481597	0.053145424	Up
HBD	14582.40103	1.040437084	0.331014946	3.14317253	0.001671273	0.057044155	Up
JUNB	724.8997753	0.985633921	0.313503113	3.143936632	0.001666915	0.057044155	Up
MIR181A2HG	466.4045896	-1.156198411	0.367624811	-3.145049997	0.001660584	0.057044155	Down
HIST1H3H	248.3734007	0.996249813	0.317263889	3.140129869	0.00168873	0.057195059	Up
HOMER2	179.5810921	1.117170356	0.355847287	3.139465716	0.001692562	0.057195059	Up
LIME1	2753.174333	-1.165701446	0.371413608	-3.138553411	0.00169784	0.057195059	Down
USP10	914.6216186	0.899605075	0.286933363	3.135240411	0.001717133	0.057594582	Up
IRAK3	712.5554955	1.037976446	0.331893827	3.127435226	0.001763387	0.058708305	Up
PGD	6221.955974	0.991085036	0.317151769	3.124955097	0.001778322	0.058708305	Up
KLRB1	2564.193431	-1.417286074	0.454542156	-3.118051991	0.001820507	0.05951598	Down

TUBB1	1929.30966	1.21262177	0.389285877	3.114990399	0.001839509	0.059633956	Up
NT5M	311.2963934	1.092784871	0.351110739	3.112365275	0.001855947	0.059667862	Up
STK33	251.7129438	0.986232209	0.317303174	3.108170007	0.001882497	0.059777009	Up
BNIP3L	378.3511888	1.025245227	0.330698301	3.100243403	0.001933617	0.060654503	Up
ESPN	201.8278341	0.967348863	0.312772236	3.092822031	0.00198263	0.061692437	Up
CDKN2D	600.8339481	0.912955306	0.295509081	3.089432317	0.002005394	0.061903555	Up
SLC26A8	173.0537996	1.03741591	0.335781138	3.089559817	0.002004533	0.061903555	Up
TSEN54	407.8446043	-0.951027312	0.308186932	-3.08587813	0.00202952	0.062399682	Down
JAK1	683.7412957	0.908125915	0.294513634	3.083476652	0.002045972	0.06241019	Up
RPL23	873.42758	-1.097524531	0.355925819	-3.083576608	0.002045284	0.06241019	Down
SLC22A15	297.0107355	0.919603857	0.300047363	3.064862324	0.002177702	0.06588633	Up
CA1	1752.047318	2.639888068	0.862595304	3.060401624	0.002210404	0.065936558	Up
FCGR1A	304.0735761	1.038498625	0.339580371	3.058182135	0.002226842	0.065936558	Up
LRWD1	646.1867132	0.944945587	0.309124655	3.056843161	0.002236813	0.065936558	Up
HINT1	4255.957854	-0.879729037	0.289265741	-3.041248629	0.002355992	0.068076239	Down
RPS23	1720.502446	-0.993638281	0.326823653	-3.040288762	0.002363514	0.068076239	Down
PGS1	2047.044657	1.220395575	0.403472535	3.024730235	0.00248855	0.069607524	Up
DCAF12	5461.632649	1.105398582	0.366853894	3.013184813	0.002585214	0.071029221	Up
BPGM	144.1175343	0.955378949	0.317389156	3.010118435	0.002611458	0.071244999	Up
PHTF1	202.407496	0.967849366	0.322520988	3.000888013	0.002691935	0.072012212	Up
RFX2	271.4441219	1.029491067	0.343106228	3.000502423	0.002695346	0.072012212	Up

SEMA4A	1714.350899	1.052350622	0.350343294	3.003769845	0.00266657	0.072012212	Up
FAM46C	6424.898486	2.257592918	0.754500042	2.9921707	0.002770013	0.072588488	Up
TLR2	265.481647	1.030604192	0.344591747	2.990797664	0.002782498	0.072588488	Up
XK	379.9923385	2.16827114	0.724743087	2.991778989	0.00277357	0.072588488	Up
CMTM5	226.012188	0.940064327	0.315356785	2.980954816	0.002873512	0.073721755	Up
ADGRG3	361.5266838	1.012121302	0.340984838	2.968229635	0.002995205	0.075346903	Up
B4GALT5	1838.837753	0.985513581	0.331925178	2.969083533	0.002986894	0.075346903	Up
GZMK	1866.216507	-1.266558036	0.428059987	-2.958833047	0.003088064	0.076543901	Down
FLOT1	2921.758078	1.12412098	0.382746141	2.936988413	0.003314165	0.078900223	Up
XPO7	628.0367908	0.882552336	0.300907917	2.932964828	0.003357419	0.079551328	Up
SESN3	869.8308394	0.910837488	0.311594569	2.92314944	0.003465102	0.081110597	Up
TBC1D22B	303.8020628	0.902439356	0.309941196	2.911647005	0.003595287	0.082659592	Up
LAPTM4B	173.2481301	0.92849265	0.319117365	2.90956479	0.003619324	0.082721296	Up
LAX1	538.8956658	-0.880123108	0.302413347	-2.91033156	0.003610455	0.082721296	Down
CLDN5	204.3414047	0.884890522	0.307520102	2.877504644	0.00400834	0.088359394	Up
DPM2	1200.906583	0.925127196	0.321754481	2.875258155	0.004036973	0.08860755	Up
GRAMD1A	1119.955345	1.218731978	0.42505403	2.867240147	0.004140687	0.090132354	Up
FFAR2	5664.016444	1.092349135	0.381146515	2.865955987	0.00415752	0.090231004	Up
FURIN	558.7023813	1.001387343	0.34963248	2.86411418	0.004181772	0.090503835	Up
SELL	11463.44892	0.993230589	0.347862575	2.855238416	0.004300453	0.092043955	Up
TSPAN9	593.0202049	1.001467461	0.351233105	2.851290058	0.004354223	0.09293807	Up

CLEC4E	254.45946	1.014509217	0.355925669	2.850340128	0.00436725	0.092960034	Up
SNHG5	963.3383075	-0.938843063	0.330360913	-2.84187089	0.004484965	0.094944014	Down
PXYLP1	171.5408091	0.912928071	0.322620311	2.829729066	0.004658744	0.096255857	Up
CD27	312.7128443	-0.932476038	0.330826914	-2.818622062	0.004823027	0.097531666	Down
CHST13	987.6830267	1.052509831	0.373420156	2.818567271	0.00482385	0.097531666	Up
FOXO3B	1149.73996	1.152364741	0.408619836	2.82013901	0.004800285	0.097531666	Up
SKAP1	581.7274915	-0.986367113	0.350649242	-2.812973747	0.004908566	0.098019506	Down
CTDSPL	493.7251813	0.94505487	0.336541429	2.808138279	0.004982883	0.098569001	Up
POR	367.204601	1.033743317	0.36837377	2.806234861	0.005012414	0.098569001	Up
AHSP	11112.66772	1.040849887	0.371850254	2.79911033	0.005124362	0.098702078	Up
GMPR	1706.062027	0.993099918	0.355316053	2.794976214	0.005190353	0.099051375	Up
MMRN1	153.9571387	0.998301395	0.358163036	2.78728203	0.00531522	0.100937066	Up
CXCR1	2786.268986	1.151775117	0.414232459	2.780504258	0.005427455	0.10096934	Up
HLA-DOA	587.9920157	-1.057269758	0.380382625	-2.779490143	0.005444431	0.10096934	Down
ANKRD22	210.1247089	1.042878423	0.375574185	2.776757471	0.005490413	0.101183987	Up
IL4R	1078.108072	1.115883953	0.403079245	2.768398442	0.005633254	0.102939744	Up
AQP10	293.1437284	1.797705804	0.652250636	2.756157995	0.005848474	0.104924438	Up
C1QB	148.5981481	-0.971635578	0.352474206	-2.756614702	0.005840313	0.104924438	Down
EPSTI1	2588.362302	1.056026493	0.386535602	2.732029051	0.006294559	0.110339917	Up
SLC2A3	8847.452018	1.015564542	0.372292935	2.727864128	0.006374586	0.110989419	Up
ODC1	1790.338662	0.928884472	0.341133809	2.722932901	0.00647052	0.111407972	Up

GYPB	623.1743647	2.060561103	0.759515674	2.712993522	0.006667841	0.112749474	Up
KCNJ2	1526.481886	1.029581395	0.379914471	2.710034693	0.006727617	0.112749474	Up
SPIB	171.75332	-0.886549452	0.326884848	-2.712115464	0.00668553	0.112749474	Down
RPL26	2419.556168	-0.887776595	0.32977612	-2.692058464	0.00710125	0.114626003	Down
NLRC4	414.6700383	1.069586546	0.399262369	2.678906479	0.007386302	0.115979561	Up
TRIM22	1332.176768	0.91934147	0.343707018	2.674782366	0.007477778	0.115979561	Up
HCAR2	356.3336713	0.978911082	0.36682167	2.668629371	0.007616144	0.11691712	Up
CD79B	1021.355197	-0.913311718	0.343416013	-2.659490774	0.007825887	0.117235994	Down
LOC101930164	196.2019301	0.953827584	0.367641275	2.59445184	0.009474189	0.129659244	Up
AP5B1	264.4064481	0.927997458	0.359864031	2.578744684	0.009916005	0.131915772	Up
CSRNP1	2029.392734	1.045997625	0.405442204	2.579893297	0.009883085	0.131915772	Up
SLC6A8	682.5992823	0.935176787	0.365657024	2.557524473	0.010542014	0.136629037	Up
BCL6	6727.264825	0.908012412	0.358473308	2.532998671	0.011309138	0.141327746	Up
H2AFJ	502.9949873	0.96108875	0.380230926	2.527644869	0.011483044	0.14280999	Up
MAOA	131.2268632	1.55952091	0.616953197	2.527778311	0.011478681	0.14280999	Up
ADM	3800.91051	0.983459472	0.390786398	2.516616437	0.011848774	0.142842215	Up
RPS18	4437.008211	-1.013940551	0.402114462	-2.521522223	0.011684829	0.142842215	Down
HIST1H2BD	333.9651271	0.912675363	0.363566446	2.510339922	0.012061499	0.144216817	Up
GRINA	2137.859201	0.896434008	0.359082659	2.496455857	0.012544128	0.147260461	Up
MCEMP1	3879.768802	2.19684457	0.881125989	2.49322412	0.012658891	0.147935281	Up
IFIT1B	1168.991673	1.925380988	0.77457726	2.485718452	0.012929021	0.149068532	Up

PTPRCAP	1155.780518	-0.935815036	0.376652245	-2.484559827	0.012971171	0.149172744	Down
IL18R1	518.2315604	2.179350663	0.893148493	2.440076515	0.014684151	0.159629687	Up
VPREB3	647.6889222	-0.93624923	0.385142052	-2.430919252	0.015060571	0.159629687	Down
DYSF	5039.127309	0.931201712	0.387236018	2.404739404	0.016184002	0.16426604	Up
IL7R	3256.778812	-0.907571972	0.381095651	-2.381480791	0.01724319	0.169093776	Down
RNF24	3371.016103	0.90472259	0.398711115	2.269118059	0.023261147	0.196326112	Up
RPS3A	4200.220935	-0.893021645	0.397214436	-2.24821045	0.024562774	0.200539906	Down
LILRA5	3187.411999	0.977533713	0.436096739	2.241552451	0.024990312	0.202113714	Up
RNASE2	2508.1251	0.889118223	0.398285379	2.232364704	0.025590869	0.204405955	Up
MMP9	3856.806563	0.896152019	0.410957996	2.180641401	0.02920995	0.216006901	Up
OLAH	176.0480277	1.748179676	0.815406697	2.143935882	0.032038025	0.226997173	Up
CASP5	445.7107141	1.580358537	0.740555856	2.134016664	0.032841417	0.229239007	Up
KRT1	976.0308412	2.030452518	0.960858184	2.11316566	0.034586591	0.234376392	Up
CMBL	184.7402771	1.255692229	0.599627389	2.094120869	0.036249214	0.23785801	Up
TPST1	413.3718624	1.743884809	0.833926346	2.09117366	0.0365125	0.238258151	Up
ARHGEF40	367.438941	1.392314035	0.667160466	2.086925269	0.03689489	0.238451502	Up
SIPA1L2	544.6099705	1.49326988	0.724233508	2.061862456	0.039220833	0.24675498	Up
MCTP2	323.0192113	1.437147595	0.699805373	2.053638985	0.040010646	0.24886686	Up
DAAM2	204.2604108	1.898213885	0.940567847	2.018157321	0.043574879	0.257920677	Up
OSM	215.2288626	1.310781738	0.659351442	1.987986459	0.046813188	0.265626609	Up

TableS 2. Characteristics of Stable Angina (SA) ,Normal and Acute Coronary Syndrome(ACS).

	**ACS** *N=30*	**Normal** *N=23*	**SA** *N=31*	**p.overall**
sex:				0.008
female	7 (23.3%)	15 (65.2%)	12 (38.7%)	
male	23 (76.7%)	8 (34.8%)	19 (61.3%)	
age(year)	64.0 [54.0;69.5]	59.0 [52.0;68.0]	68.0 [61.0;72.5]	0.063
BMI	23.9 (3.32)	23.2 (3.09)	24.3 (3.45)	0.517
hypertension:				<0.001
No	13 (43.3%)	19 (82.6%)	7 (22.6%)	
Yes	17 (56.7%)	4 (17.4%)	24 (77.4%)	
diabetes:				0.536
No	24 (80.0%)	21 (91.3%)	25 (80.6%)	
Yes	6 (20.0%)	2 (8.70%)	6 (19.4%)	
smoking_history:				0.005
No	14 (46.7%)	20 (87.0%)	15 (48.4%)	
Yes	16 (53.3%)	3 (13.0%)	16 (51.6%)	
WBC(*10^9/L)	11.8 [10.2;13.2]	5.59 [4.12;6.27]	6.28 [5.44;7.54]	<0.001
N（%）	86.9 [78.9;89.3]	59.4 [57.2;66.8]	62.7 [58.8;69.4]	<0.001
Mono.per(%)	4.23 (2.20)	5.99 (1.97)	5.95 (2.09)	0.002
Mono(*10^9/L)	0.36 [0.27;0.60]	0.31 [0.23;0.49]	0.35 [0.30;0.44]	0.411
Platelet(*10^9/L)	211 (30.5)	228 (58.4)	207 (51.5)	0.261
PDW(fL)	16.0 [12.3;16.4]	13.1 [11.8;15.2]	13.1	0.069

	ACS<br>*N=30*	Normal<br>*N=23*	SA<br>*N=31*	p.overall
			[11.8;16.0]	
MPV(fL)	9.80 [9.30;10.4]	10.5 [9.90;11.1]	10.3 [9.45;10.9]	0.040
PCT(ng/ml)	0.21 [0.19;0.22]	0.23 [0.21;0.28]	0.21 [0.19;0.24]	0.042
P-LCR (%)	25.1 [20.3;27.8]	28.1 [23.4;33.5]	27.3 [21.8;32.8]	0.138
GLU(mmol/L)	6.12 [5.54;7.22]	5.01 [4.44;5.50]	5.48 [4.94;6.78]	0.002
UA(μmol/L)	366 [320;429]	298 [248;356]	328 [292;392]	0.081
BNP(pg/ml)	126 [37.6;350]	35.1 [25.9;47.7]	40.4 [23.2;66.8]	0.001
cTn(ng/L)	17654 [8705;83448]	2.90 [1.90;4.80]	5.20 [2.15;18.2]	<0.001
CK(U/L)	689 [277;2060]	85.0 [58.5;116]	80.0 [60.0;116]	<0.001
CK-MB(U/L)	75.0 [31.2;207]	14.0 [11.5;16.5]	12.0 [10.0;14.5]	<0.001
Cr(μmol/L)	72.0 [53.8;82.5]	60.0 [51.0;68.5]	71.0 [60.0;81.5]	0.006
HCY(μmol/L)	9.61 [8.54;13.3]	10.2 [8.35;11.6]	10.4 [8.71;13.0]	0.650
CRP(mg/L)	2.50 [1.31;20.2]	1.50 [0.90;3.05]	1.21 [0.90;3.71]	0.062
TC(mmol/L)	4.76 [3.96;5.34]	4.63 [4.06;5.62]	4.25 [3.38;5.48]	0.512
TG(mmol/L)	1.45 [0.84;1.94]	1.54 [1.10;2.16]	1.69 [1.52;2.10]	0.164
HDL(mmol/L)	1.00 [0.81;1.25]	1.13 [1.00;1.35]	1.01 [0.90;1.13]	0.081

	ACS	Normal	SA	p.overall
	N=30	*N=23*	*N=31*	
LDL(mmol/L)	2.71 (0.74)	2.79 (0.88)	2.54 (0.96)	0.560
TBIL(μmol/L)	11.4 [8.75;15.4]	11.4 [7.40;13.1]	9.10 [6.80;11.0]	0.026
IBIL(μmol/L)	7.90 [5.93;11.3]	7.80 [4.95;9.25]	6.40 [4.45;7.45]	0.017
DBIL(μmol/L)	3.15 [2.65;5.48]	2.70 [2.20;3.70]	2.60 [2.45;3.40]	0.104
CD147_OD_platelet	1.10 (0.19)	1.00 (0.29)	0.77 (0.28)	<0.001
CD147_OD_Mono	0.96 [0.92;1.05]	1.00 [0.91;1.08]	0.95 [0.91;1.07]	0.917

Continuous data conforming to a normal distribution were reported as mean (SD) and those not conforming as median(quartiles). Statistical significance was determined using t-test, Rank-sum test or χ^2 test, with p-values calculated.

SA: Stable Angina. ACS: Acute Coronary Syndrome. BMI: body mass index. WBC: white blood cell. N: neutrophil. Mono.Per: monocytes percentage. mono: monocytes. PDW: platelet distribution width. MPV: Mean Platelet Volume. PCT: procalcitonin. P-LCR: platelet -larger cell ratio. GLU: Glucose. UA: uric acid. cTn: cardiac troponin. CK: creatine kinase. CK-MB: creatine kinase-MB. Cr: creatinine. HCY: homocysteine. CRP: C-reactive protein. TC: total cholesterol. TG: Triglycerides. HDL: high-density lipoprotein. LDL: low-density lipoprotein. TBIL: total bilirubin. IBIL: indirect bilirubin. DBIL: direct bilirubin.

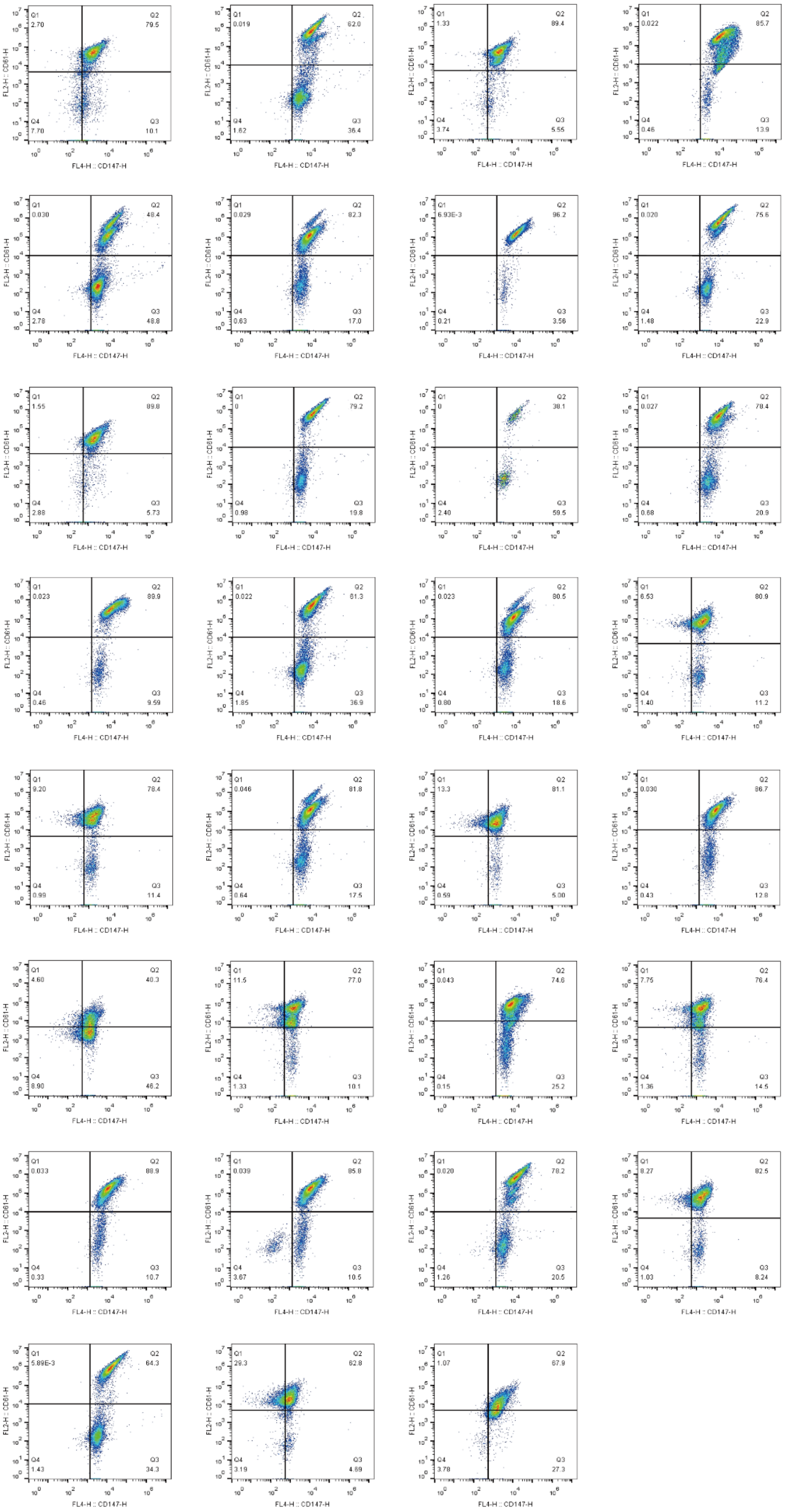
FL2-H :: CD61-H
FL4-H :: CD147-H

FigureS 1. Flow Cytometric Platelet Analysis of 31 Patients with Stable Angina Pectoris.

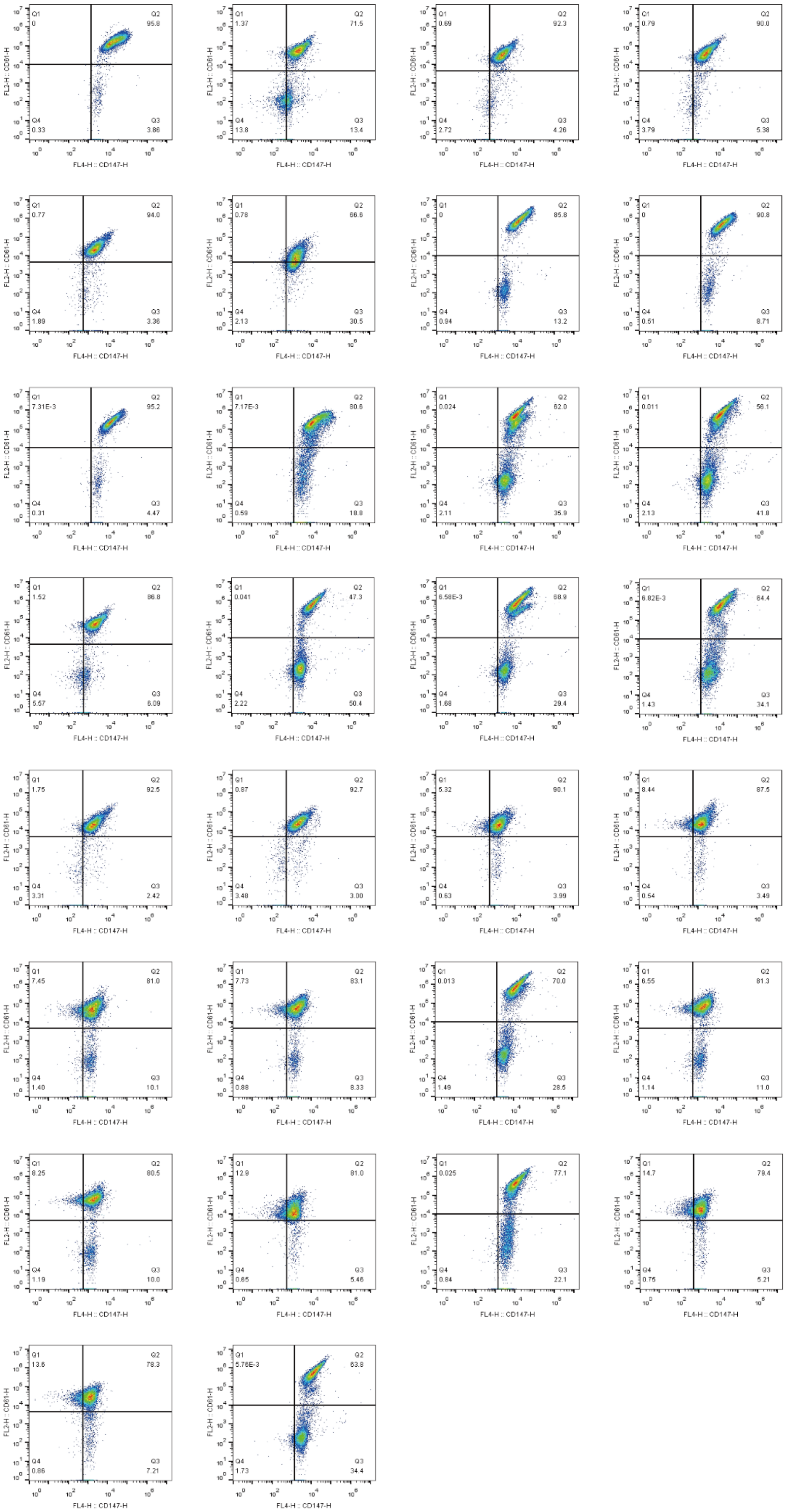

FL2-H :: CD61-H
FL4-H :: CD147-H
Q1 0
Q2 95.8
Q4 0.33
Q3 3.86
Q1 1.37
Q2 71.5
Q4 13.8
Q3 13.4
Q1 0.69
Q2 92.3
Q4 2.72
Q3 4.26
Q1 0.79
Q2 90.0
Q4 3.79
Q3 5.38
Q1 0.77
Q2 94.0
Q4 1.89
Q3 3.36
Q1 0.78
Q2 66.6
Q4 2.13
Q3 30.5
Q1 0
Q2 85.8
Q4 0.94
Q3 13.2
Q1 0
Q2 90.8
Q4 0.51
Q3 8.71
Q1 7.31E-3
Q2 95.2
Q4 0.31
Q3 4.47
Q1 7.17E-3
Q2 80.6
Q4 0.59
Q3 18.8
Q1 0.024
Q2 62.0
Q4 2.11
Q3 35.9
Q1 0.011
Q2 56.1
Q4 2.13
Q3 41.8
Q1 1.52
Q2 86.8
Q4 5.57
Q3 6.09
Q1 0.041
Q2 47.3
Q4 2.22
Q3 50.4
Q1 6.58E-3
Q2 68.9
Q4 1.68
Q3 29.4
Q1 6.82E-3
Q2 64.4
Q4 1.43
Q3 34.1
Q1 1.75
Q2 92.5
Q4 3.31
Q3 2.42
Q1 0.87
Q2 92.7
Q4 3.48
Q3 3.00
Q1 5.32
Q2 90.1
Q4 0.63
Q3 3.99
Q1 8.44
Q2 87.5
Q4 0.54
Q3 3.49
Q1 7.45
Q2 81.0
Q4 1.40
Q3 10.1
Q1 7.73
Q2 83.1
Q4 0.88
Q3 8.33
Q1 0.013
Q2 70.0
Q4 1.49
Q3 28.5
Q1 6.55
Q2 81.3
Q4 1.14
Q3 11.0
Q1 8.25
Q2 80.5
Q4 1.19
Q3 10.0
Q1 12.9
Q2 81.0
Q4 0.65
Q3 5.46
Q1 0.025
Q2 77.1
Q4 0.84
Q3 22.1
Q1 14.7
Q2 79.4
Q4 0.75
Q3 5.21
Q1 13.6
Q2 78.3
Q4 0.86
Q3 7.21
Q1 5.76E-3
Q2 63.8
Q4 1.73
Q3 34.4

FigureS 2. Flow Cytometric Platelet Analysis of 30 Patients with Acute Coronary Syndrome.

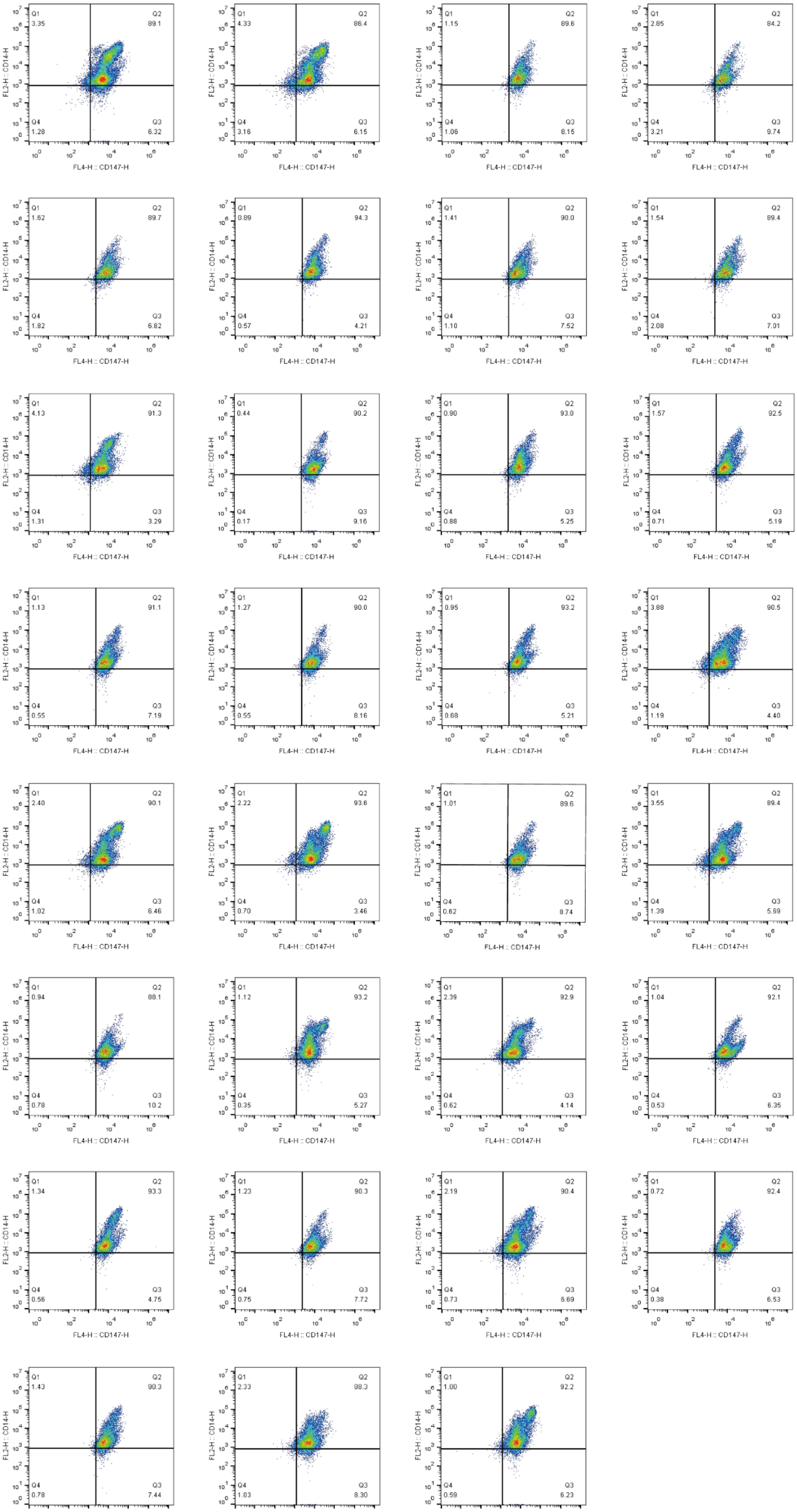
FL2-H :: CD14-H
FL4-H :: CD147-H
Q1 3.35 Q2 89.1 Q4 1.28 Q3 6.32
Q1 4.33 Q2 86.4 Q4 3.16 Q3 6.15
Q1 1.15 Q2 89.6 Q4 1.06 Q3 8.15
Q1 2.85 Q2 84.2 Q4 3.21 Q3 9.74
Q1 1.62 Q2 89.7 Q4 1.82 Q3 6.82
Q1 0.89 Q2 94.3 Q4 0.57 Q3 4.21
Q1 1.41 Q2 90.0 Q4 1.10 Q3 7.52
Q1 1.54 Q2 89.4 Q4 2.08 Q3 7.01
Q1 4.13 Q2 91.3 Q4 1.31 Q3 3.29
Q1 0.44 Q2 90.2 Q4 0.17 Q3 9.16
Q1 0.90 Q2 93.0 Q4 0.88 Q3 5.25
Q1 1.57 Q2 92.5 Q4 0.71 Q3 5.19
Q1 1.13 Q2 91.1 Q4 0.55 Q3 7.19
Q1 1.27 Q2 90.0 Q4 0.55 Q3 8.16
Q1 0.95 Q2 93.2 Q4 0.68 Q3 5.21
Q1 3.88 Q2 90.5 Q4 1.19 Q3 4.40
Q1 2.40 Q2 90.1 Q4 1.02 Q3 6.46
Q1 2.22 Q2 93.6 Q4 0.70 Q3 3.46
Q1 1.01 Q2 89.6 Q4 0.62 Q3 8.74
Q1 3.55 Q2 89.4 Q4 1.39 Q3 5.69
Q1 0.94 Q2 88.1 Q4 0.78 Q3 10.2
Q1 1.12 Q2 93.2 Q4 0.35 Q3 5.27
Q1 2.39 Q2 92.9 Q4 0.62 Q3 4.14
Q1 1.04 Q2 92.1 Q4 0.53 Q3 6.35
Q1 1.34 Q2 93.3 Q4 0.56 Q3 4.75
Q1 1.23 Q2 90.3 Q4 0.75 Q3 7.72
Q1 2.19 Q2 90.4 Q4 0.73 Q3 6.69
Q1 0.72 Q2 92.4 Q4 0.38 Q3 6.53
Q1 1.43 Q2 90.3 Q4 0.78 Q3 7.44
Q1 2.33 Q2 88.3 Q4 1.03 Q3 8.30
Q1 1.00 Q2 92.2 Q4 0.59 Q3 6.23

FigureS 3. Flow Cytometric Monocyte Analysis of 31 Patients with Stable Angina Pectoris.

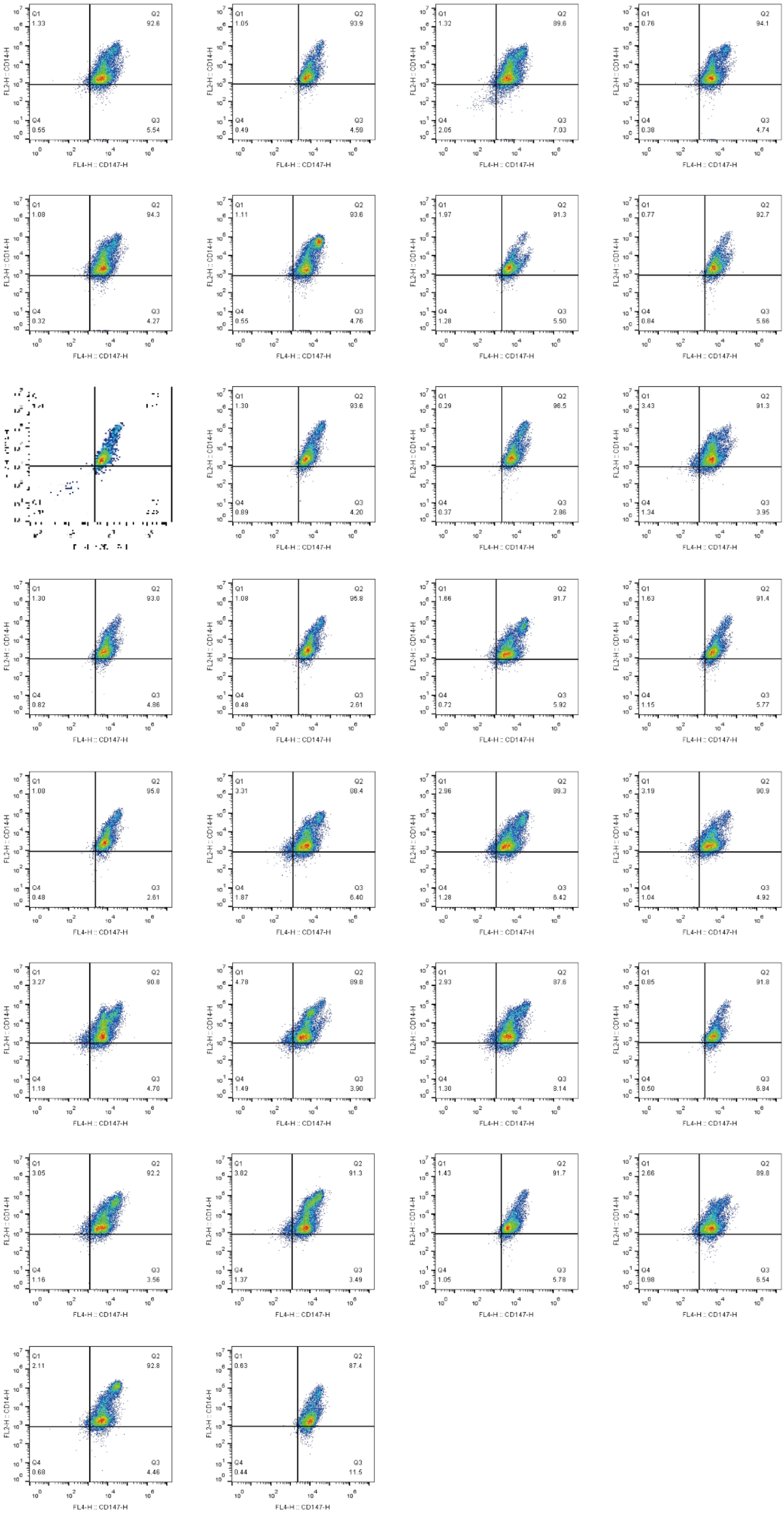
FL2-H :: CD14-H
FL4-H :: CD147-H
Q1
Q2
Q3
Q4

FigureS 4. Flow Cytometric Monocyte Analysis of 30 Patients with Acute Coronary Syndrome.
